# Criteria for referring pediatric and adult patients with hematological diseases to palliative care: Consensus of the Brazilian Association of Hematology, hemotherapy and cell therapy (2025)

**DOI:** 10.1016/j.htct.2026.106456

**Published:** 2026-04-24

**Authors:** Amanda Pifano Soares Ferreira, Cecília Emerick Mendes Vaz, Laura Ferreira de Mesquita Ferraz, Paulo de Mello Novita Teixeira, Mariana Munari Magnus, Clarissa de Miranda Fonseca, Júlia Alvarenga Petrocchi, Suzana de França Ribeiro Gonzaga, Tathiana Rodrigues Peres Braz, Rita de Cássia Rosário Cavalheiro, Yara Andrea Pires Afonso Reina, Sandra Regina Loggetto, Lorena Lobo de Figueiredo Pontes, Sílvia Maria Meira Magalhães, Renato Sampaio Tavares, Carlos Sérgio Chiattone, Vânia Tietsche de Moraes Hungria, Phillip Scheinberg, Carmino Antônio de Souza, Fernando Ferreira Costa, Paula de Melo Campos

**Affiliations:** a Grupo Oncoclínicas, Belo Horizonte, Brazil; b Instituto de Oncologia Ciências Médicas de Minas Gerais, Belo Horizonte, Brazil; c Grupo Oncoclínicas, Rio de Janeiro, Brazil; d Amil Assistência Médica, Rio de Janeiro, Brazil; e Hospital de Base do Distrito Federal, Brasília, Brazil; f Santa Casa de Misericórdia de Ourinhos, Ourinhos, Brazil; g Centro de Hematologia e Hemoterapia, Universidade Estadual de Campinas – Hemocentro (Unicamp), Campinas, Brazil; h Hospital de Apoio de Brasília, Brasília, Brazil; i Unimed Sorocaba, Sorocaba, Brazil; j Centro de Hematologia de Sorocaba, Sorocaba, Brazil; k Grupo Oncoclínicas, São Paulo, Brazil; l Hospital Santa Marcelina, São Paulo, Brazil; m Oncologia D’Or, São Paulo, Brazil; n Sabará Hospital Infantil, São Paulo, Brazil; o Banco de Sangue São Paulo – GSH, São Paulo, Brazil; p Divisão de Hematologia, Hemoterapia e Terapia Celular, Departamento de Imagens Médicas, Hematologia e Oncologia Clínica, Faculdade de Medicina de Ribeirão Preto, Universidade de São Paulo, Ribeirão Preto, Brazil; q Serviço de Hematologia, Hospital Universitário Walter Cantídio, Universidade Federal do Ceará, Fortaleza, Brazil; r Hospital das Clínicas da Universidade Federal de Goias (HC UFG), Goiania, GO, Brazil; s Faculdade de Ciências Médicas da Santa Casa de São Paulo (FCMSCSP), São Paulo, SP, Brazil; t Serviço de Hematologia, Centro de Oncologia e Hematologia da Beneficência Portuguesa de São Paulo, São Paulo, Brazil

**Keywords:** Palliative care, Symptom burden, Quality of life, Advance care planning, Hematology

## Abstract

Palliative care is, according to the World Health Organization (WHO), “a global ethical responsibility” and crucial to person-centered healthcare, for the relief of physical and psychological symptoms and social and spiritual suffering, impacting the improvement of the quality of life not only of pediatric and adult patients dealing with life-threatening illnesses, but also of their families. Despite the growing recognition of palliative care in the hematological setting and the increase in the number of publications on the subject, the available data are still scarce and limited. The objective of this consensus is to establish recommendations for the referral of pediatric and adult hematological patients to specialized palliative care teams and the possible integration of these two specialties.

## Introduction

In 1990, the World Health Organization (WHO) published the first definition of Palliative Care (PC). Subsequently, in 1998, it addressed the topic with a particular focus on children, and since then, this issue has undergone several reformulations and updates. According to a revision in 2018, the WHO states that PC is “an approach that improves the quality of life (QoL) of patients (adults or children) and their families facing problems associated with life-threatening illness. PC prevents and alleviates suffering through early investigation, correct assessment, and treatment of pain and other physical, psychosocial, or spiritual problems.” [1].

It is important to emphasize that QoL is a subjective assessment that goes beyond the concern of eradicating diseases and their symptoms, as it is also directly related to the effects caused by health interventions on all aspects of an individual's life, within their culture, the value systems in which they live, and in relation to their goals, expectations, standards, and concerns [2].

Complementing the WHO definition, the International Association for Hospice and Palliative Care (IAHPC) stated that PC is active care, especially, but not only, for those in the final stages of life [3]. Given its inherent complexity, PC should be delivered by a multidisciplinary team comprising a minimum core of specialized professionals, including a physician, nurse, psychologist, or social worker. The interdisciplinary framework may further incorporate other specialists, such as nutritionists, physiotherapists, occupational therapists, and chaplains, among others [1]. The composition of the team and the duties and responsibilities of its members vary according to the patient's needs, available resources, and the social context.

Evidence shows that early intervention with PC significantly improves QoL, symptoms, and satisfaction of patients with advanced cancer, and may even prolong survival [4,5]. Despite this evidence, it is well known that the integration of PC in hematological diseases has lagged behind that of solid oncology for numerous reasons. The stigmatized association between this treatment and end-of-life care is responsible for many patients with advanced hematological diseases still not receiving PC [6]. Routinely, data already consolidated for solid tumors are extrapolated to hematology, [4,7] and, knowing that the trajectories and prognostic estimates are quite different in these scenarios, this model of reasoning is an obstacle to the integration of PC services. Prognostic uncertainty and the use of intensive treatments, which can result in a cure while still presenting a high risk of mortality, make it difficult to define referral criteria for hematological malignancies (HM) and end-of-life indicators in hematology [8,9].

The availability of effective and safe targeted therapies and immunotherapies for a variety of HM has increased the expectation of cure; however, this is sometimes at the cost of significant morbidity and distressing symptoms, since a significant proportion of patients will not respond to or tolerate these new therapies [10,11]. Estimating prognosis and envisioning outcomes are challenging when faced with new therapeutic possibilities; they also reduce the chances of discussing the risks and benefits of a new treatment, addressing care preferences, and defining the care plan for the end-of-life phase.

Most patients with HM want to participate in decision-making regarding their treatments, but consider the information they receive to be insufficient or unclear, as exemplified in different studies by the significant discrepancy between the expectations of doctors and patients regarding both treatment responses and chances of cure, as well as diagnostic understanding and disease trajectory [12, 13, 14]. Patients with a high risk of mortality, but who maintain a small chance of cure, should be informed about possible outcomes, from the best to the worst scenarios. Early integration of PC is recognized as an ideal way to allow patients and families to be more aware of the possible prognoses and cope with the psychosocial sequelae and uncertainties inherent in therapies, enabling greater patient involvement in decisions about their treatment [8,11,15,16]. Ultimately, the unique and close bond between hematologists and their patients, often rooted in deep trust and strong clinical rapport developed throughout the treatment process, can create barriers to the integration of other professionals into the care team [17].

In an attempt to define the optimal timing for the integration of PC and to contribute to care planning, Shaulov et al [18] proposed specific trajectories for hematological diseases, categorizing them into three distinct groups:

a) aggressive diseases (e.g., acute leukemias, aggressive lymphomas) and bone marrow transplantation: life-threatening diseases with a high risk of morbidity associated with the possibility of long-term treatment, often with cure being a reasonable and achievable goal until days before death;
b) indolent diseases (e.g., chronic lymphocytic leukemia, indolent lymphomas, and multiple myeloma [MM]): incurable diseases with progressive loss of functionality due to multiple relapses and different lines of treatment, associated with periods of prolonged remission and preserved performance status;
c) bone marrow failure (e.g., myelodysplastic syndrome, myelofibrosis, aplastic anemia [AA]): a group characterized by cytopenias secondary to bone marrow failure, leaving the patient dependent on blood transfusions and susceptible to recurrent bacterial and fungal infections, in which the prognosis is challenging as death results from acute complications of cytopenias, and not from disease progression [18].

**Fig. 1:**
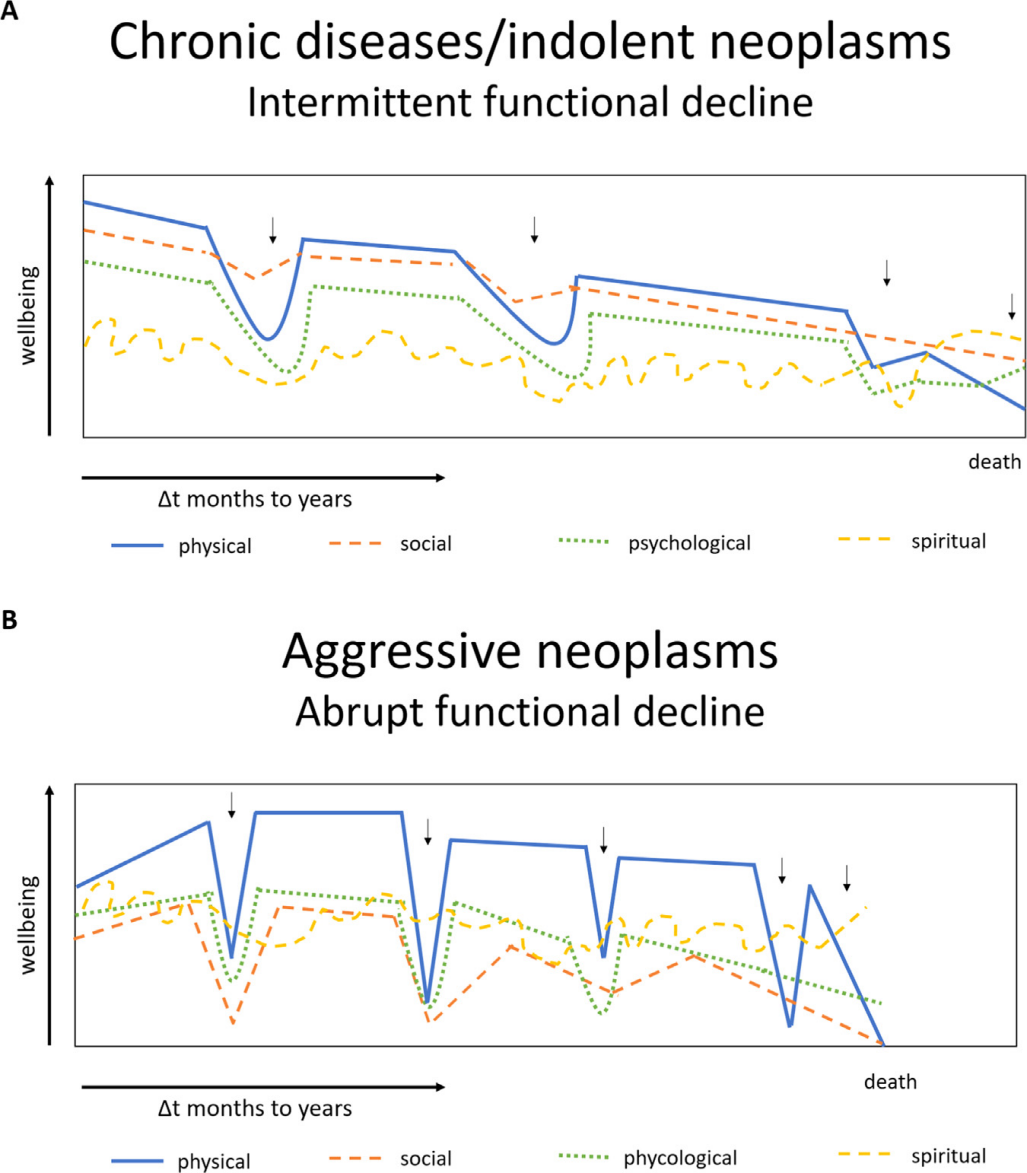
Trajectories of hematological diseases across the four dimensions of care: physical, social, psychological, and spiritual.

Based on an adaptation of these models and the publication by Murray et al., [19] we suggest the following trajectories for benign and malignant hematological diseases (Figure 1).

(A) Indolent hematological neoplasms, bone marrow failure, and benign chronic diseases – patients in this group are frequently followed for months to years in hematology services, possibly with alternating periods of disease-modifying treatment and periods of expectant management. During follow-up, various acute events (small arrows) (e.g., hospitalizations, infections, hemorrhages, worsening anemia, etc.) or the accumulation of comorbidities resulting from chronic disease combined with aging can lead to a worsening of physical and psychological symptoms. At these times, impairment of social capacity is frequent, while spirituality is variable, depending on the characteristics of each individual. After the resolution of acute events, incomplete recovery of health conditions is common, with progressive loss of functionality over time. The development of prognostic awareness deepens over the follow-up period as functionality is lost. (B) Aggressive hematological neoplasms (acute leukemias, high-grade lymphomas) and bone marrow transplantation – in addition to the manifestations caused by the neoplasm itself, patients in this group are frequently subjected to aggressive treatments, resulting in potentially life-threatening acute complications (small arrows) (e.g., sepsis, hemorrhages, gastrointestinal symptoms, tumor lysis, etc.), which can lead to abrupt loss of functionality in the physical, social, and psychological dimensions, with or without subsequent recovery. Spirituality is variable, depending on the individual characteristics of the patients. Given the aggressiveness of the HM in this group, the mortality rate within weeks to months is substantial, and establishing earlier prognostic awareness is fundamental.

Fortunately, there has been an increase in the number of publications on PC in hematology, demonstrating the impact that this partnership can generate in all dimensions of suffering [20, 21, 22]. Following this trend, the 2024 update of the American Society for Clinical Oncology (ASCO) guidelines recommends referral to PC specialists for all patients with HM [23].

Despite the growing recognition of PC and the number of publications in the literature, including randomized trials that confirm the benefits of PC integrated with HM, the available data are still scarce and limited, especially when considering benign diseases. The objective of this consensus is, therefore, to establish criteria that encourage the possible integration and referral of pediatric and adult hematological patients to a specialized PC team, seeking a joint action for the benefit of patients in their entire environment, including their support network and healthcare team.

## Specifics in pediatrics

Influenced by the *hospice* movement in adults, pediatrics has adopted a more holistic approach, transforming and expanding awareness of multidimensional suffering in children and their families. Pediatric and adolescent patients cannot be considered separately, since their families are directly or indirectly involved in patient care, and thoughts about the possibility of death and the end of life vary among them. Care and communication should be patient-centered and family-focused [24,25]. Most pediatricians aspire to promote the physical and emotional well-being of their patients' development, hoping to offer a long and healthy life, but a portion of cases will not achieve this.

In 1979, Smith et al. described an approach that provided total care for children with cancer and their families, aiming to meet physical, emotional, and financial needs [26,27]. In 1983, Lewis argued that the principles of alleviating suffering should be applied to all seriously ill children as ongoing care, and not only to those who are dying [26,28,29]. Until then, the management of pain and end-of-life symptoms, which were limited to one paragraph, began to be studied by small groups of specialists with the collaboration of parents, aiming at improving care [26].

In general, children and adolescents undergo painful procedures such as bone marrow aspiration, lumbar punctures, and venous catheter insertions, both at diagnosis and during treatment, and sometimes after remission. This exposes them not only to a high burden of physical symptoms, but also psychological, social, and spiritual ones. Chemotherapy protocols, in most cases, do not facilitate the outpatient administration of medications, and due to age, side effects, bureaucratic issues, or complications, patients remain hospitalized for long periods, isolated from their family, school, and friends. Visual and bodily changes, especially in adolescence, secondary to hair loss and weight gain, impact self-esteem, triggering greater social isolation, depressive episodes, and anxiety. Denial, or the desire to be like other adolescents, can lead patients to engage in risky behaviors and abandon medical treatments, with serious consequences. No less important, as they become young adults, they must leave the familiar environment of pediatric doctors and nurses who have cared for them so far in their lives and establish a relationship with an unfamiliar care team, with health care often being transferred from parents to the patients themselves [25,30].

Studies show that children with advanced cancer, including those with HM, are more likely to receive intensive end-of-life treatment compared to adults. Furthermore, they are more likely to receive intravenous chemotherapy (CT) in the 14 days preceding death, even in advanced stages of the disease [31]. Mortality rates in patients with malignant HM are higher than those with solid tumors due to treatment-related complications. Consequently, there are higher rates of admission to the pediatric intensive care unit (ICU) for acute respiratory failure, septic shock, and severe hemorrhage [31]. Due to the more effective response to CT, there is a high number of different protocols, including for relapse, resulting from technological advances in specific therapies, the possibility of hematopoietic stem cell transplantation (HSCT), and chimeric antigen receptor T cell (CAR-T) therapy, making prognostic estimation and the decision-making process more complex [31,32].

A study analyzed PC opportunities in different childhood cancer diagnosis groups and identified that children with solid tumors and central nervous system tumors have more opportunities for PC compared to those with leukemia or lymphoma [33]. This suggests that early identification of PC opportunities can guide the use of these services to alleviate the stress of patients and their families [33]. PediQUEST, an important trial in children with advanced cancer, evaluated the use of a computerized tool for assessing patient-reported outcomes (PROs) [34]. The primary objective was to verify whether providing feedback to healthcare providers and families with respect to PROs could improve health-related QoL and reduce suffering and symptom burden. The secondary objective was to assess the level of agreement between parents and healthcare providers regarding patient outcomes. Children with advanced cancer aged two years or older, or their parents, completed the PediQUEST questionnaire weekly, including adapted versions of the Memorial Symptom Assessment Scale (MSAS) and the Pediatric QoL Inventory 4.0 (PedsQL4.0), and a general question about the disease [35]. While there was no feedback in the control group, in the intervention group, oncologists and family members received printed reports summarizing the PROs, and emails were sent to oncologists when certain scores were exceeded. The results showed that feedback did not significantly affect the mean trends in MSAS and PedsQL4.0 scores or disease progression [34]. However, exploratory analyses suggested improvements in emotional QoL in specific subgroups, such as children aged eight years or older who survived for 20 weeks. Furthermore, feedback from PROs was valued by children, parents, and providers, contributing to the initiation of psychosocial consultations in indicated cases. The importance of strategies to overcome the ‘normalization’ and undervaluation of symptoms, which prevent the use of appropriate interventions to alleviate suffering in childhood cancer, was also highlighted. This support is crucial for improving QoL and can be integrated by PC teams [36,37].

## Methods

This consensus was written by hematologists specializing in PC, members of the PC Committee of the Brazilian Association of Hematology, Hemotherapy and Cell Therapy (ABHH), in collaboration with members of the respective specialty committees of the ABHH. The criteria of the Grading of Recommendations Assessment, Development and Evaluation (GRADE) [38] working group were used to define the levels of evidence and strength of the recommendations. Since scientific production on PC in hematological diseases is limited, the levels of evidence for the recommendations are weak. Therefore, to construct referral criteria that are in accordance with the principles of PC and the particularities of each group of hematological diseases, the recommendations were developed by the PC committee and established after discussion with specialists in each area covered in this Consensus through the respective specialty committees of the ABHH.

This document was developed after a systematic search of scientific articles related to the topic. References were searched in databases (PubMed, SciELO, Web of Science, ScienceDirect, Latin American and Caribbean Literature [LILACS]., and SCOPUS) with no limit on publication date or format. The following keywords were used: “palliative care”, “hospice care”, “symptom management”, “symptom burden”, “quality-of-life”, “end-of-life”, “caregiver outcome”, “advance care planning”, “treatment decision-making”, “health service utilization”, “place of death”, “prognostic tools”, “prognostic scores”, “predictors”, “acute leukemia”, “lymphoma”, “lymphoproliferation”, “multiple myeloma”, “amyloidosis”, “myelodysplastic syndrome”, “myelofibrosis”, “myeloproliferative neoplasm”, “chronic myeloid leukemia”, “essential thrombocythemia”, “polycythemia vera”, “bone marrow transplant”, “CAR-T cells”, “sickle cell”, “thalassemia”, “hemoglobinopathies”, “hemophilia”, “thrombosis”, “bone marrow failure”, “neutropenia”, “pediatrics”, and “children”. Articles with abstracts related to the theme of this consensus were evaluated in full.

### Palliative care in hematology—General criteria of referral

According to a 2015 publication by the American Society of Hematology (ASH), referral to a specialized PC team should be considered in some general scenarios, regardless of the patient's diagnosis and age.

### Recommendations

#### General criteria for referring hematological patients to palliative care

1. Patients with a high symptom burden or symptoms refractory to established treatments; impaired QoL and significant psychological distress (mood disorders or existential suffering); sequelae and late side effects related to established treatments. (Level of recommendation: B)
2. Patients with difficulty understanding diagnosis and prognosis; those with significant or persistent misperceptions about the trajectory of their disease and overall prognosis. (Level of recommendation: B)
3. Patients experiencing difficulties coping with their illness; need for communication adjustments; patient involvement in the shared decision-making process and in defining end-of-life care preferences; assistance in conflict resolution. (Level of recommendation: D)
4. Support for distressed family members/caregivers. (Level of recommendation: D)
5. Reserved prognosis or limited life expectancy (surprise question: "Would you be surprised if your patient died within 1 year?"). (Level of recommendation: B)
6. Patients with recurrent unplanned hospitalizations and loss of performance status/functionality. (Level of recommendation: D)

### Palliative care in hematology—Referral criteria according to diagnosis

• Acute leukemias

Acute leukemias are malignant clonal disorders of hematopoietic stem cells, classified according to the origin of the abnormal cells. They are among the most distressing cancer diagnoses, because very soon after receiving the diagnosis, most patients will face a prolonged hospital stay, experiencing a short transition between feeling healthy and having a life-threatening illness [39].

Acute myeloid leukemia (AML) is a predominantly adult disease, with a median age at diagnosis of 68 years; [26] it accounts for approximately 15 % of acute childhood leukemias [31,40]. In pediatrics, only one-third of patients achieve remission and are cured, with an overall five-year survival rate of 82 % for those classified as low risk, while high-risk patients still have very poor outcomes, with a survival rate of approximately 46 % [31,40]. Acute promyelocytic leukemia (APL) and AML associated with Down syndrome have favorable prognoses [41]. In the adult age group, with standard treatment (intensive chemotherapy ‘7 + 3’), long-term survival for AML patients is 35-45 % for patients younger than 60 years and 10-15 % for those older than 60 years [26]. Despite many recent advances, the prognosis for over-60-year-old patients remains poor, with up to 70 % of patients aged 65 or older dying within one year of diagnosis [26]. Newer regimens and targeted therapies have reduced treatment-related toxicity and increased remission rates, offering hope to a greater proportion of older patients with AML. However, these approaches have not significantly improved long-term cure rates, often extending median survival only by weeks or months [42,43]. This was demonstrated by the VIALE-A clinical trial (Venetoclax and Azacitidine), which reported a median overall survival of 14.7 months [44].

Acute lymphoblastic leukemia (ALL) is traditionally considered a pediatric disease, as it is the most common and prevalent neoplasm in this age group. Even so, about half of all diagnoses occur in approximately equal proportions in younger adults (18-49 years) and older adults (50 years and older) [30]. ALL accounts for 30 % of malignancies between 0 and 14 years of age, with a high incidence especially in children aged 1 to 4 years, representing about 75-80 % of acute leukemias. ALL presents significant racial and ethnic disparities, including lower five-year survival rates in black children [40]. These disparities may be influenced by socioeconomic factors, access to healthcare, and biological differences in disease presentation. Patients with Down syndrome have a greater predisposition to infections and, therefore, need to be treated with reduced CT regimens [40,45]. In adolescents and young adults (15-39 years old), ALL is still prevalent, but the survival rate is lower compared to under-15-year-old patients, reflecting not only the biological complexity but also differences related to treatment [40,45]. The follow-up of adolescents and young adults with ALL presents a significant challenge due to non-adherence to treatment regimens and high rates of absenteeism from appointments, related to emotional factors (such as depression and low self-esteem), patient health beliefs, and their family environment [46]. Unfortunately, the high cure rates seen in children have not been replicated in older cohorts. Young adults have over 70 % long-term survival with the use of protocols inspired by pediatric treatments, while older adults (aged 55 to 60 years or older) have a less than 20 % cure rate [46]. Older adults, in addition to comorbidities, present greater toxicity secondary to CT, treatment delays, high rates of early mortality, and treatment-related mortality in those in remission [46,47]. The main unfavorable prognostic factors are positive minimal residual disease and relapsed or refractory (R/R) disease. Immunotherapy has emerged as a revolutionary form of treatment, often reducing the need for allogeneic HSCT, [40] but despite advances, a portion of patients will not be cured [31,48].

To better understand the burden of symptoms presented by patients diagnosed with acute leukemia, Zimmermann et al [49] followed patients with newly diagnosed disease or recent relapse, demonstrating a median of nine physical symptoms (range: 0-22) and two psychological symptoms (range: 0-6), among which the most prevalent were: fatigue (79 %), drowsiness (56 %), xerostomia (54 %), weight loss (54 %), and pain (49 %). The most prevalent psychological symptoms were insomnia (55 %), worry (43 %), difficulty concentrating (39 %), and sadness (36 %). Fatigue, insomnia, and pain were the most intense symptoms, with those presenting them being more likely to report intense worry and sadness [49]. A substantial drop in QoL scores (measured by the Patient Care Monitor v2.0 (PCM), the Functional Assessment of Cancer Therapy-Leukemia [FACT-Leu], and the National Comprehensive Cancer Network [NCCN] distress thermometer [DT]) was observed in a cohort of AML patients within the first two weeks of starting induction CT. These scores did not return to baseline levels by Week 4, with the greatest reductions occurring in physical and functional well-being [50]. Depressive symptoms such as hopelessness and sadness were reported by 15 % and 16 % of patients, respectively [50]. To minimize the burden of disease-related symptoms, a randomized phase II clinical trial evaluated an intervention named ‘Emotion and Symptom-Focused Engagement’ (EASE). This intervention was delivered in a hospital setting to patients with a recent diagnosis of acute leukemia, specifically within one month of their initial diagnosis [51]. This intervention integrates supportive psychotherapy combined with trauma-focused cognitive behavioral therapy (CBT), in addition to systematic screening for physical symptoms. Patients were referred early for PC when symptom intensity reached a score of 4 or greater. The results demonstrated that an integrated intervention for the control of psychological and physical symptoms led to significant reductions in symptoms of traumatic stress and acute stress disorder (DSM IV criteria), pain intensity, and pain interference in daily activities, when compared with usual care [51]. Additional secondary outcomes, including the severity of physical symptoms and resulting distress, depressive disorders, satisfaction with care, and overall QoL, showed non-statistically significant trends favoring the intervention group [51].

Aiming to evaluate the early integration of PC in hospitalized patients with AML receiving intensive CT, a randomized clinical trial was conducted as follows: patients were seen by the PC team (physician or nurse) at least twice per week starting within 72 h of randomization and followed up during all subsequent hospitalizations for up to one year. The results showed that patients seen by the PC team experienced substantial improvements in QoL, with a reduction in symptoms of depression, anxiety, and post-traumatic stress disorder (PTSD) during hospitalization, with the impacts lasting up to six months after diagnosis. Moreover, the patients who died were more likely to discuss their end-of-life care preferences with their physicians and were also less likely to receive chemotherapy in the last 30 days of life. To investigate the effect of PC interventions on coping with the diagnosis among patients with AML, Nelson et al [53] reported that the joint action of the PC team facilitated a greater use of coping strategies (acceptance, positive reframing, and emotional support), while simultaneously reducing the reliance on avoidance strategies (behavioral disengagement, denial, and self-blame). These changes in coping were associated with improvements in QoL and a reduction in symptoms of depression and anxiety in Week 2 of treatment, a time when patients are presumed to feel worse at the nadir of CT [53]. Another randomized controlled trial evaluated the impact of monthly outpatient PC, supplemented by at least two visits per week during hospitalization, in a cohort of 115 patients with high-risk AML and Myelodysplastic Syndromes (MDS) undergoing non-intensive CT [54]. Patients who were followed by the PC team showed higher rates of discussion and documentation of end-of-life care preferences, a longer interval between documentation and death, and a greater propensity to discuss care with their attending physicians [54]. There was a reduction in the hospitalization rate in the last 30 days of life, and, in three months of follow-up, patients showed better QoL, with no differences in the depression and anxiety rates [54].

### Recommendations

#### Criteria for referring patients with acute leukemia to palliative care

1. AML patients aged 18 years or older receiving intensive chemotherapy in a hospital setting: Early integration of PC with admission by the PC team within 72 h of the start of chemotherapy and at least two visits per week during hospitalization and subsequent hospitalizations. (Level of recommendation: A)
2. AML patients receiving non-intensive chemotherapy: PC integrated early with admission by the PC team within 30 days of treatment initiation, with at least one monthly outpatient visit, and at least twice a week during each hospital stay. (Level of recommendation: A)
3. ALL patients aged 18 years or older receiving intensive chemotherapy in a hospital setting: extrapolating the evidence from PC to AML patients, we suggest early integration of PC with admission by the PC team within 72 h of the start of chemotherapy and at least two visits per week during hospitalization and subsequent hospitalizations. (Level of recommendation: D)
4. Pediatric patients with AML or ALL: Extrapolating the evidence to patients aged 18 years or older with AML, we suggest early integration of PC with admission by the PC team within 72 h of the start of chemotherapy and at least two visits per week during hospitalization and subsequent hospitalizations. (Level of recommendation: D)
5. Newly diagnosed patients with acute leukemia requiring only best supportive treatment. (Level of recommendation: D)
6. Patients with acute leukemia and a high symptom burden, refractory symptoms, moderate to intense psychological or existential distress, in end-of-life care, or presenting any of the general criteria for referral of hematological patients to PC. (Level of recommendation: D)

## Myelodysplastic neoplasms

MDS comprise a heterogeneous group of myeloid disorders characterized by ineffective erythropoiesis, morphological cellular dysplasia in the bone marrow, cytopenias in peripheral blood, and a risk of progression to AML [55]. They affect men more frequently from the seventh decade, [56] with the main risk factors being a history of chemotherapy or radiotherapy and exposure to chemical agents such as benzene [57]. In adults and the elderly, the most common manifestation is anemia, with the bone marrow evaluation generally being hypercellular. Children diagnosed with MDS may present with frequent bleeding and infections resulting from severe thrombocytopenia and neutropenia; isolated anemia is less common. A bone marrow evaluation may show hypocellular bone marrow. As MDS is a diagnosis of exclusion, patients should be evaluated for secondary causes [40].

Both the MDS Revised-International Prognostic Scoring System (IPSS-R) [58] and the more recent Molecular International Prognostic Scoring System (IPSS-M), which incorporates molecular data into risk classification [59], are utilized to stratify the risk of leukemic transformation and estimate overall survival. For low-risk groups, the estimated median survival is up to ten years, with most deaths unrelated to leukemic transformation. In high-risk groups, there is a high rate of progression to AML in a short period of time, with a median overall survival of three years [56]. Patients with low-risk MDS may present with minimal symptoms and may not require CT or serial transfusions, and may receive specific therapies targeted at clinically relevant cytopenias, such as transfusion support, growth factors, immunomodulators (e.g., lenalidomide), transforming growth factor-beta (TGF-β) neutralizers (e.g., luspatercept), and immunosuppressants (e.g., cyclosporine). Patients with high-risk MDS experience high morbidity and mortality rates, high transfusion demands, bleeding, recurrent infections, and progression to AML, with a very unfavorable prognosis. Treatment with disease-modifying drugs aims to improve overall survival, such as the use of hypomethylating agents, intensive chemotherapy, and HSCT, the only potentially curative treatment, with disease-free survival rates between 30–50 %. Determining the best treatment for these patients takes into account age, comorbidities, clinical performance, availability of treatments, and a suitable bone marrow donor [60, 61, 62].

Patients with MDS present a high symptom burden with consequent impairment of health-related QoL [61]. Lack of recognition of these symptoms can lead to inadequate management, potentially reducing patient functionality and consequently impacting adherence to and tolerability of disease-modifying and supportive treatments [61]. The symptoms most frequently reported by patients are fatigue (89 %), insomnia, daytime sleepiness, and dyspnea [63]. The estimated incidence of frequent bleeding, bone pain, unwanted weight loss, and recurrent infections is 55 %, 39 %, 25 %, and 20 %, respectively [64]. Although fatigue is a constitutional symptom that correlates weakly with anemia rates, it significantly compromises QoL. Specifically, it impairs work capacity and reduces participation in leisure activities [65]. Patients who develop transfusion dependence experience up to a 50 % reduction in QoL scores [66]. A retrospective study of patients with HM, 67 % of whom had a diagnosis of MDS, was conducted to evaluate the impact of early CP on symptom control. This study demonstrated that, after the fourth CP intervention visit, there was a statistically significant reduction in pain, depression, anxiety, and anorexia [67]. An evaluation of an extensive retrospective database of over seven thousand elderly patients with MDS demonstrated that 28 % of patients were admitted to the ICU and that transfusion dependence correlated with lower rates of transfer to hospices and higher rates of ICU admission in the end-of-life phase [68]. The main causes of death among patients with MDS relate to transformation to AML (46 %), infection (27 %), and bleeding (10 %); other common causes are heart failure and secondary hemochromatosis [69].

A randomized clinical trial that included high-risk AML and MDS patients showed that patients who received early integrated PC had a longer time between the documentation of care preferences and death, were more likely to discuss their end-of-life care preferences, and had lower rates of hospital admission in the last 30 days of life, with a significant improvement in QoL [54].

### Recommendations

#### Criteria for referring patients with myelodysplastic syndromes to palliative care

1. Patients with high-risk MDS receiving non-intensive chemotherapy: Early integration of PC with admission by the PC team within 30 days of starting treatment, with at least one monthly outpatient visit, and at least twice a week during each hospital stay. (Level of recommendation: A)
2. Patients with MDS receiving intensive chemotherapy in a hospital setting: extrapolating the evidence from PC to patients with AML, we suggest early integration of PC with admission by the PC team within 72 h of the start of chemotherapy, and at least two visits per week during hospital stays. (Level of recommendation: D)
3. Patients with intermediate-high, high-risk, and very high-risk MDS who are indicated for best supportive care only. (Level of recommendation: D)
4. Pediatric patients diagnosed with MDS: we suggest early integration of PC, regardless of the chosen treatment strategy. (Level of recommendation: D)
5. Patients with MDS and a high symptom burden, refractory symptoms, moderate to intense psychological or existential distress, or the presence of any of the criteria described in the general referral criteria of hematological patients to PC. (Level of recommendation: D)

## Chronic myeloproliferative neoplasms

Chronic myeloproliferative neoplasms (CMPNs) constitute a heterogeneous group of diseases affecting one or more myeloid lineages, resulting from aberrant clonal proliferation. The current WHO classification subdivides the group into seven categories based on distinct clinical, molecular, and prognostic characteristics, but with a burden of symptoms and complications that often overlap [70]. Studies in clinical practice divide them into two major groups: chronic myeloid leukemia (CML) and Philadelphia-negative CMPNs.

CML is rare in childhood, with an incidence in children and adolescents of less than 5 % [45]. In Western countries, the average age of CML patients is around 57 years [61]. Patients with chronic phase CML treated with tyrosine kinase inhibitors (TKIs) usually have a mean survival similar to the general population [71,72]. However, the good prognosis coexists with the prospect of long-term treatments resulting in repercussions on physical, psychosocial, and financial conditions, [71,73,74] which contribute to the manifestation of depression, anxiety, and lack of self-recognition by the patient [75]. Fatigue is the most frequent and most intense physical symptom (51 %), but nausea, diarrhea, cramps, and muscle pain are also common [71,72,75,76]. Discontinuation of TKIs in patients with deep molecular responses appears to result in symptom control and improvement in QoL in young patients, but with minimal effect in over 60-year-old patients [77]. It is estimated that 25 % of patients may need to switch TKIs due to resistance or intolerance, with an increasing rate of therapeutic failure as subsequent line changes become necessary [78]. A suboptimal response to two or more CT treatments should prompt immediate consideration of HSCT.

Patients with advanced CML at diagnosis should be treated as high-risk, becoming eligible for HSCT if response to CT is not optimal. Progression to the accelerated phase has become a rare event, and HSCT should be considered immediately if it occurs during treatment [79]. Blastic crises maintain a guarded prognosis with survival generally less than one year due to bleeding or infections; [76] the treatment plan is like that of acute leukemias, culminating in allogeneic HSCT when disease remission occurs [79]. In an analysis of Medicare end-of-life care, it was found that, among patients with CML, 52 % were in the ICU in the last 30 days of life and 22 % received CT within the last 14 days [80]. Hospice care significantly reduced these numbers to 25 % and 9 %, respectively, as well as healthcare spending in the last 30 days of life (US$31,400 versus US$ 18,500), [80] suggesting a potential benefit of integrating PC into the usual care of patients with CMPNs.

Among Philadelphia chromosome-negative MPNs, the most common are Polycythemia Vera (PV), Essential Thrombocythemia (ET), and Primary Myelofibrosis (PM). Fatigue is the predominant symptom, reported in 99 %, 92 %, and 90 % of patients, respectively [81]. This symptom significantly impacts functionality and social life, often requiring assistance with work and activities of daily living [82,83]. Other recurrent symptoms include pruritus, night sweats, early satiety, thrombosis, bleeding, and sexual dysfunction, with a high incidence of symptoms related to distress (40 %), anxiety (31 %), and depression (12.5-23 %) [84]. Proactive symptom assessment is recommended at all consultations, preferably using validated tools such as the Myeloproliferative Neoplasm Symptom Assessment Form Total Symptom Score (MPN-SAF TSS) [85]. With proven clinical applicability, the MPN-SAF TSS is a questionnaire that measures symptom burden in patients with MPN using a numerical scale from 1 to 10 (a score of 7 or higher indicates severe symptoms) [86]. The frequency varies according to the MPN subtype, although consistently, patients with MF had the highest symptom burden [87].

Patients with MF have a life expectancy that can vary from months to years, so risk stratification models seek to refine the prognosis and assist in decision-making, especially regarding the timing of allogeneic HSCT. Despite currently being associated with a rate of at least 30 % of procedure-related deaths or severe morbidity, HSCT is the only treatment modality capable of prolonging survival with curative potential [88]. The International Prognostic Scoring System (IPSS), Dynamic International Prognostic Scoring System (DIPSS), and the Refined Dynamic International Prognostic Scoring System (DIPSS-plus) are considered adequate to characterize the prognosis of these patients [70]. The median survivals according to the DIPSS-plus risk categories range from 15.4 years (low risk) to 1.3 years (high risk). More recently, molecular assessment has been integrated into the development of three new prognostic models for myelofibrosis (MF): the Mutation-Enhanced International Prognostic Score System for Transplantation-Age Patients (MIPSS70), the Mutation and Karyotype-Enhanced International Prognostic Scoring System version 2.0 (MIPSS70+ v2.0), and the Genetically Inspired Prognostic Scoring System (GIPSS) [88].

The evolution of PV and ET to secondary myelofibrosis is part of the natural history of these diseases, with an average transformation time estimated to be between 7 and 10 years [89]. The rate of transformation to AML within the first decade following diagnosis varies between 10 % and 20 % in patients with MF, while it is approximately 2.3 % in those with PV and less than 1 % in ET; notably, such transformation is associated with a dismal prognosis with average survival ranging from 1.5-2.5 months when on supportive treatment, and 3.9-9.4 months among those who receive intensive treatment [90]. The three-year survival rate after HSCT varies between 16 and 33 % [90].

### Recommendations

#### Criteria for referring patients with chronic myeloproliferative neoplasms to palliative care

1. Patients with chronic phase CML who have failed or are intolerant to two or more TKIs or have progressed to an advanced phase and are candidates for allogeneic HSCT. (Level of recommendation: D)
2. Patients with CML blast crisis or CMPN progressing to acute leukemia on an intensive chemotherapy treatment strategy: extrapolating the PC evidence to patients with AML, we suggest early integration of PC, with admission by the PC team within 72 h of the start of chemotherapy and at least two visits per week during hospitalizations. (Level of recommendation: D)
3. Patients diagnosed with primary myelofibrosis and DIPSS-plus intermediate-2 and high-risk. (Level of recommendation: D)
4. Patients diagnosed with progression from PV or ET to secondary myelofibrosis. (Level of recommendation: D)
5. Patients with CMPN and an indication for only best supportive treatment. (Level of recommendation: D)
6. Patients with CMPN and high symptom burden, refractory symptoms, moderate to intense psychological or existential distress, or the presence of any of the criteria described in the general referral criteria of hematological patients to PC. (Level of recommendation: D)

## Lymphoproliferative neoplasms

Lymphoproliferative neoplasms constitute a heterogeneous group of malignant diseases of the lymphatic tissue with a broad prognostic spectrum. There are more than 60 types and subtypes of lymphomas according to the histological classification [91]. The third most common cause of death in children and adolescents, [40] lymphomas represent about 4 % of all cancers and 3.3 % of cancer deaths in the USA [92]. In Brazil, it is estimated that more than five thousand deaths occur annually due to lymphoma [92]. They are classified as Hodgkin's lymphoma (HL) and Non-Hodgkin lymphoma (NHL) and develop from the accumulation of acquired somatic mutations, with viral infections (Epstein-Barr, hepatitis C, and HIV) among their main risk factors, although, for most cases, there is no evident etiological factor [93].

HL is most common among people aged 15 to 40, with a second peak in incidence after 60 years old. The combination of CT, radiotherapy, and monoclonal antibodies allows for a cure in about 80 % of HL patients under 60 years of age [93]. When relapse or refractoriness occurs, patients should receive high-intensity CT followed by autologous HSCT, which can be challenging in elderly and frail patients [94].

NHLs account for approximately 60 % of lymphoma cases. The classic division into high-grade (aggressive) and low-grade (indolent) lymphomas is found in the 2022 classification and helps to interpret clinical behavior and define the need for treatment. Differences in biological behavior, pathophysiological mechanisms, and cytogenetic/molecular alterations result in very varied prognoses [91]. High-grade lymphomas have a high rate of cell replication and growth, presenting symptoms such as pain, superior vena cava compression syndrome, fatigue, weight loss, and fever. Most types are sensitive to chemotherapeutic and immunotherapeutic treatments, with potential for cure. In patients with aggressive R/R lymphomas, prognostic uncertainty is exacerbated by recent therapeutic advances and the increasing attainability of a cure [95]. Indolent lymphomas, on the other hand, are characterized by slow and progressive growth and may not require immediate treatment, but are managed using a ‘watch and wait’ strategy. They are considered an incurable disease with periods of clinical remission interspersed with periods of relapse, a high symptom burden during disease activity, and the need for subsequent lines of treatment with a progressive accumulation of toxicities [95]. The rapid changes in the clinical conditions of patients and the possibility of functional recovery, even in advanced disease scenarios, pose a challenge to prognostic definition and the consequent early referral to PC [96]. Patients present high rates of pain, dyspnea, fatigue, depression, and intense and limiting pruritus, all of which tend to worsen near the end of life [95,97]. Although disease-modifying therapies have shown exponential evolution in the last decade, a significant proportion of patients still die from disease progression and refractoriness or suffer from uncontrolled physical and emotional symptoms [98].

In childhood, the most common NHLs are Burkitt lymphoma, lymphoblastic lymphoma, and diffuse large B-cell NHL, which exhibit aggressive or very aggressive behaviors [40]. The primary clinical presentation consists of mediastinal widening, with or without associated pleural effusion, which may rapidly progress to superior vena cava syndrome. This condition constitutes an oncological emergency and may occur alongside infiltration of the bone marrow and the central nervous system (CNS) [40,99,100]. In the adult population, the most common subtype of high-grade NHL is diffuse large B-cell NHL. The complete response rate after treatment with CT and monoclonal antibodies is 70 %, with event-free survival around 50 %. However, 10 % of patients will be primarily refractory, and between 30 and 40 % will experience disease relapse.[78]. For these R/R patients, five-year survival is less than 50 %, even after salvage chemotherapy and autologous HSCT. CAR-T cell therapy has emerged as a potential alternative to induce prolonged remissions or even curative treatment in a subgroup of patients [101]. A study with 267 patients with diffuse large B-cell NHL evaluated the relationship between sarcopenia and prognosis. The findings showed that patients with an intermediate and low sarcopenia index (CXI) had a worse prognosis compared to those with a high CXI, [102] suggesting that this score may be a patient-related prognostic biomarker, reflecting the impact of frailty syndrome on the survival of patients with lymphoma, and a potential referral criterion for PC [103].

Although there are few specific studies of PC in patients with lymphoma, research that has evaluated hospice-associated lymphoma, including lymphomas, has demonstrated a reduction in futile end-of-life interventions, improved alignment of medical goals with care preferences of patients, and increased referral to hospice-type end-of-life care [104, 105, 106]. Referral of patients with lymphomas to PC should be less related to prognosis and more to patient needs (e.g., in aggressive T-cell R/R lymphomas, the likelihood of a durable response is typically even more somber than for B-cell lymphomas, and predictors of sustained response are unclear) [77]. Refractoriness of symptoms, emotional distress, difficulty understanding the disease, the need for complex discussions regarding care goals, and the possibility of serious complications or death within one year should be triggers for PC referral [98].

### Recommendations

#### Criteria for referring patients with lymphoproliferative malignancies to palliative care

1. Pediatric and adult patients with R/R lymphomas. (Level of recommendation: D)
2. Adult patients with aggressive lymphoproliferative malignancies and frailty syndrome, low functionality, or sarcopenia. (Level of recommendation: B)
3. Pediatric and adult patients with lymphoproliferative malignancies and an indication for best supportive care only. (Level of recommendation: D)
4. Pediatric and adult patients with lymphoproliferative malignancies and a high symptom burden, refractory symptoms, moderate to intense psychological or existential distress, or the presence of any of the criteria described in the general referral criteria of hematological patients to PC. (Level of recommendation: D)

## Monoclonal gammopathies

Among the Monoclonal Gammopathies, there are Monoclonal Gammopathies of Undetermined Significance (MGUS), asymptomatic or smoldering MM (MMS), and symptomatic MM, the latter being of greatest interest due to the high prevalence of symptom burden, mainly pain, and the incurable nature of the disease, directly impacting QoL and causing an immense psychological burden even in its stable phases [107, 108, 109].

A cross-sectional analysis of patients with MGUS and MMS assessed symptom burden and psychological distress; the results were not significantly different between participants, and, to a certain point, similar to those found in previous studies of patients with MM [110]. The prevalence of anxiety, depression, and symptoms of PTSD were 27 %, 14 %, and 24 %, respectively. Overall, 31 % of patients reported moderate to severe symptom burden and the presence of fatigue, malaise, drowsiness, and pain. It is not possible to conclude whether the described physical symptoms are attributable to MGUS or MMS [110].

The median age at diagnosis of MM is 69 years, and associated with a high prevalence of frailty and comorbidities. This demographic profile increases the challenges regarding treatment and symptom management, as the symptom burden is often greater in both frequency and intensity than in other HM [107]. Due to population aging associated with the availability of new therapies, there is a significant upward trend in the global incidence among the elderly, from 6.7 million in 2012 to an expected 14 million in 2035 [107,108]. A study in Denmark showed that 50 % of patients have symptoms considered intense, with high levels of pain, fatigue (98 %) and constipation (66 %) Patients also have problems related to physical function and social role, with high rates of depression (26 %) and anxiety (23 %), and low scores in functional scales [111,112]. Symptoms are related to the underlying disease, treatment-related side effects, and cumulative toxicity, particularly in the context of disease relapse or continuous therapy. Consequently, patients endure prolonged periods of physical symptoms and psychological dysfunction, which may lead to treatment discontinuation and, subsequently, disease progression and compromised clinical outcomes [112,113]. Furthermore, family members are shown to be as psychologically burdened as the patients stemming from extensive caregiving responsibilities related to medication, mobility, and assistance with activities of daily living [109].

In the literature, there are few recommendations regarding PC in MM, with most focusing on symptom control: bone pain, anemia, peripheral neuropathy, constipation, infection, and thrombosis prophylaxis [114]. It is believed that PC should be integrated as soon as possible, according to service availability, mainly with the aim of controlling clinical signs and symptoms and other comorbidities that interfere with the patient's well-being [115,116]. In a retrospective analysis, 55 patients with MM received early PC, while 231 received usual hematological care [117]. Patients managed by the PC team experienced superior pain control, characterized by the prolonged use of strong opioids and higher rates of symptom management, as well as greater psychological support and more frequent and earlier discussions regarding goals of care. Furthermore, there was a trend toward fewer aggressive end-of-life interventions, including anti-MM treatment within 14 to 30 days of death, and cardiopulmonary resuscitation, or orotracheal intubation within the last 30 days. Notably, these patients also had fewer hospitalizations exceeding seven days, and fewer than two hospital admissions or emergency department visits in their final 30 days, while maintaining equivalent survival rates [117].

Porta-Sales analyzed 67 patients referred to a PC clinic with an average time of 355 days between diagnosis and first consultation. The study demonstrated a significantly lower proportion of patients with moderate to severe pain; less interference of pain in general activities, sleep, and mood; and a lower rate of depression [116]. A multicenter study evaluated 557 patients in various stages of the disease: 1) newly diagnosed (pre-treatment or first-line treatment); 2) treatment-free interval (stable disease with no evidence of disease progression); and 3) relapsed/progressive disease (second-line or higher-level therapy or progression in treatment). In this study, the most prevalent symptoms were pain, fatigue, memory changes, shortness of breath, peripheral neuropathy, and reduced mobility. Among the concerns regarding QoL were "problems performing usual activities," "concern that the disease might worsen," and "not having enough information about what might happen in the future." [118]. The burden of symptoms and concerns about QoL is greater in those with relapsed/progressive disease due to the fear of manifesting previous symptoms, followed by those with newly diagnosed disease, who worry about what might happen, the impact of the new treatment, and the occurrence of side effects. Symptom scores tend to be higher in patients receiving treatment due to the presence of side effects associated with underlying comorbidities; however, those with stable disease also face a multitude of physical and psychosocial challenges, as many live with chronic pain, peripheral neuropathy, or associated organ dysfunctions, in addition to the fear of relapse [118]. When the disease becomes refractory to available therapies, early care planning should focus on transitioning from drug treatment to symptom control and clarifying end-of-life care preferences [115].

Another important monoclonal gammopathy is systemic light chain (AL) amyloidosis, which can culminate in rapidly progressing, often devastating, multi-organ dysfunction through amyloid deposits [119]. Clinical manifestations are deceptive and sometimes recognized only at an irreversible stage with an adverse prognosis. Complications include heart failure, hypotension, pleural effusions, renal involvement, including nephrotic syndrome with peripheral edema, gastrointestinal symptoms leading to anorexia and cachexia, complex pain syndromes, and mood disorders [120]. Cardiac involvement has the greatest impact on determining survival in these patients, followed by renal involvement [121]. Patients and their families should have a realistic understanding of the disease and the goals and limitations of treatments so that they can make informed decisions about medical therapy and supportive care, as well as plan for end-of-life care. Given such a complex presentation and high morbidity and mortality rates, establishing a PC program in clinical management is a means to improve QoL, reduce emergency room visits, readmissions, and hospital mortality [120].

### Recommendations

#### Criteria for referring patients with monoclonal gammopathies to palliative care

1) Patients with symptomatic MM or medullary/extramedullary plasmacytoma: patients with moderate to severe physical or psychosocial symptoms at any stage of the disease; moderate to severe treatment-related side effects or toxicity, and impaired QoL. (Level of recommendation: D)
2) Patients with systemic AL amyloidosis: patients with moderate to severe physical or psychosocial symptoms at any stage of the disease; moderate to severe side effects or toxicity related to treatment, and impaired QoL. (Level of recommendation: D)
3) Patients with MM or systemic AL amyloidosis and an indication for only best supportive treatment. (Level of recommendation: D)
4) Patients with MM or systemic AL amyloidosis and high symptom burden, refractory symptoms, moderate to intense psychological or existential distress, or the presence of any of the criteria described in the general referral criteria of hematological patients to PC. (Level of recommendation: D)

## Bone marrow transplantation and cell therapy

### Hematopoietic stem cell transplantation

HSCT is an intensive and potentially curative therapy for various hematological diseases, both malignant and non-malignant. Its high toxicity and morbidity are fully justified by the ultimate goal: the pursuit of restoring a normal life free from the underlying disease. It is divided into two modalities: (1) autologous, where the source of the graft is the patient's own stem cells collected before the conditioning protocol; and (2) allogeneic, in which the stem cells come from a donor [74]. There are several differences between pediatric and adult HSCT recipients. These include the biology of the diseases; variations related to age and body size; body composition; physiological differences in organ function and metabolism; and psychosocial factors affecting family dynamics, neurodevelopment, and resource availability [99]. Prolonged hospitalizations and social isolation are associated with decreased patient-reported QoL, elevated levels of anxiety and depressive symptoms, including pronounced anhedonia [74]. Despite its clear indication, it is a procedure with significant risks, including side effects from conditioning chemotherapy, cytopenias, and acute and chronic graft-versus-host disease (GvHD) [122, 123, 124].

Studies suggest that the intensity of physical symptoms experienced during HSCT may be a factor in greater psychological distress over time, [125] with psychological stressors also being associated with higher risks of GvHD and decreased overall survival [74]. GvHD is generally a mild condition, but it can sometimes be fatal. Psychosexual dysfunction is common in HSCT recipients, as are neuro-hormonal toxicities related to chemotherapy, negative body image, and financial toxicity [126]. The presence of PTSD symptoms is commonly described [127,128]. According to the guidelines of the American Society for Transplantation and Cellular Therapy (ASTCT), [99] early consultation with the PC team is essential to establish a multidisciplinary relationship and discuss care goals, including pain management and coping strategies [32,41].

A randomized study comparing standard treatment (HSCT alone; n = 79) with integrated care (HSCT combined with twice-weekly PC consultations; n = 81) demonstrated that the integrated approach resulted in a significantly smaller decline in QoL from baseline to week two, that is, the peak symptomatic period. Furthermore, patients receiving integrated care experienced a lower increase in symptom burden and significantly lower rates of depression and anxiety compared to the standard treatment group [22]. Three months after HSCT, patients maintained higher QoL scores and lower rates of depressive symptoms [22]. These data align with those described by El-Jawahri et al., with lower rates of depression and PTSD six months after HSCT, with clear benefits for survivors [129]. In line with this study, the literature highlights the importance of early integration of PC in pediatric transplants, [41,48,130,131] highlighting the high receptivity by patients and their parents, and the perception that attention to QoL should be a priority from the beginning of treatment [48,130,131].

An exploratory analysis of two randomized clinical trials 22, 132 evaluated the specific factors associated with the magnitude of patient response to CP intervention. Participants were categorized as either ‘high’ or ‘low’ responders based on the variation in their QoL scores from admission to two weeks after HSCT. The analysis revealed that belonging to a racial minority, having a lower baseline QoL, and experiencing a higher burden of physical symptoms or PTSD were all associated with a greater likelihood of being a high responder. Furthermore, these high-responding patients reported significantly greater use of coping strategies at the two-week, three-month, and six-month marks post-HSCT [133].

Regarding the clinical consultations themselves, high-responder visits placed a heavier emphasis on disease education, HSCT-related information, and support for coping and symptom control. In contrast, visits for low-responding patients focused predominantly on symptomatic management. These findings suggest that a CP approach to education and coping reduces disease-related uncertainty, thereby improving QoL. Notably, no differences in response were observed regarding the underlying disease, the type of HSCT, or the conditioning regimen used [133].

Caregivers of patients undergoing HSCT face a prolonged and intense caregiving burden, which negatively impacts QoL, physical well-being, and mood. In the pre-HSCT period and during the procedure, suffering is high, as their loved one experiences treatment of toxicities, physical and psychological symptoms, prolonged hospitalization, and prognostic uncertainty [74]. In the post-HSCT period, home settings will presumably require caregivers to assume greater functional demands and social problems, such as isolation, financial demands, and family tension, compromising their well-being [134].

Current evidence points to the need for integration between HSCT and PC services; however, specialized PC interventions evaluated in the included clinical trials were limited to the inpatient setting and were designed to improve QoL during hospitalization after HSCT. However, events experienced by the patient after HSCT, such as GvHD, infections, and disease recurrence, also negatively affect QoL [133]. There is still no ideal format described in the literature for establishing this partnership, but it is already known, through well-designed studies, that the presence of PC services in HSCT units contributes to the lasting relief from suffering for both patients who progress to end-of-life care and survivors [22,52,124].

### Cell therapy (chimeric antigen receptor T cells)

Cell therapy is based on the use of CAR-T cells: genetically modified T cells capable of recognizing and fighting a specific tumor [105]. In Brazil, this treatment is approved by the National Health Surveillance Agency (ANVISA) for the following R/R HM with the potential to induce prolonged remissions and be a curative alternative for this subgroup of patients: diffuse large B-cell NHL, B-cell ALL, follicular NHL, and MM [105]. Although it presents good tolerability, the toxicity of CAR-T therapy can appear up to 30 days after the infusion, [135] with the most serious and frequent toxicities being cytokine release syndrome (CRS) and CAR-T-related encephalopathy [136]. CRS can appear between the first and twelfth day after infusion, with severe cases (Grade III or IV toxicity) in 20 % of patients [105,136]. Both SLC and CAR-T therapy-related encephalopathy are reversible if managed appropriately, with deaths rarely documented [137,138]. Secondary neoplasms after CAR-T therapy represent a small fraction of reported adverse events, suggesting an increased risk for myeloid and T-cell malignancies [139].

To date, there are few studies evaluating the benefits of partnering with PC teams. However, given the nature of the procedure, which is very similar to that of HSCT, its indications in R/R diseases, the prognostic uncertainty, and the resulting need for symptom management and psychosocial support, we believe it is possible to extrapolate the indications for PC in HSCT to CAR-T. The involvement of the PC team in this setting, in addition to facilitating communication between teams, patients, and families, also enables the planning of care objectives [99].

### Recommendations

#### Referral criteria for patients undergoing hematopoietic stem cell transplantation or chimeric antigen receptor T cell therapy for palliative care

1. Patients aged 18 years or older admitted for HSCT: PC integrated early, with admission by the PC team within 72 h of hospital admission and at least two visits per week during hospitalization and subsequent hospitalizations. (Level of recommendation: A)
2. Pediatric patients hospitalized for HSCT: extrapolating data from the adult population, we suggest early integration of PC, with admission by the PC team within 72 h of hospital admission and at least two visits per week during hospitalization and subsequent hospitalizations. (Level of recommendation: D)
3. Adult and pediatric patients with Grade II to IV GvHD: outpatient follow-up by the PC team. (Level of Evidence D)
4. Adult and pediatric patients hospitalized for CAR-T cell therapy: extrapolating the evidence from PC for HSCT, we suggest early integration with admission by the PC team within 72 h of hospital admission and at least two visits per week during hospitalization and subsequent hospitalizations. (Level of recommendation: D)
5. Adult and pediatric patients eligible for CAR-T cell therapy may be referred to outpatient PC for care plan alignment. (Level of Evidence D)
6. Adult and pediatric patients undergoing HSCT or CAR-T cell therapy with a high symptom burden, refractory symptoms, moderate to intense psychological or existential distress, or the presence of any of the criteria described in the general referral criteria of hematological patients to PC. (Level of recommendation: D)

## Congenital bone marrow failure, aplastic anemia, and paroxysmal nocturnal hemoglobinuria

Bone marrow failure syndromes encompass a heterogeneous group of diseases characterized by bone marrow insufficiency, resulting in an inadequate production of blood cells and consequent isolated cytopenias or pancytopenia. These conditions may have an immune (acquired) etiology, as occurs in idiopathic AA, or a genetic (congenital/hereditary) etiology as in Fanconi anemias, congenital dyskeratosis, and telomeropathies, among others. Although they share the same hematological phenotype – failure of hematopoiesis – the pathophysiological mechanisms are distinct, being autoimmune in acquired forms and related to genetic defects in cell maintenance or repair in congenital forms [140].

Hereditary forms are usually identified in childhood and adolescence, but may only be diagnosed in adulthood [40,140]. Fanconi anemia, a progressive bone marrow failure in the presence of multiple congenital abnormalities, is predisposed to transformation into acute leukemias, MDS, and head and neck tumors [40,140, 141, 142]. Dyskeratosis Congenita is a rare disease with progressive bone marrow failure and is recognized by a classic triad: skin hyperpigmentation, nail dystrophy, and leukoplakia [40,143]. Shwachman-Diamond syndrome manifests as exocrine dysfunction, skeletal abnormalities, cytopenias, and possible progression to MDS and acute leukemias [143,144]. Although the only treatment is HSCT, the results are unsatisfactory [40,143]. Blackfan Diamond anemia is a congenital red blood cell hypoplasia that occurs in early childhood; approximately 50 % of cases present with congenital physical abnormalities, absence of red blood cell precursors, and a predisposition to cancer. Treatment up to one year of age is limited to blood transfusions and corticosteroid therapy; however, in the absence of response, allogeneic HSCT is indicated [143,145].

Congenital amegakaryocytic thrombocytopenia (CAMT), a severe thrombocytopenia that occurs from birth, is a diagnosis of exclusion. It does not usually respond to corticosteroids, immunoglobulin, or splenectomy, and HSCT is indicated in cases where platelet counts are lower than 50 × 10^9^/L [146]. Among severe neutropenias, Kostmann syndrome is characterized by severe congenital neutropenia with treatment generally involving the use of granulocyte colony-stimulating factors (G-CSF) to increase neutrophil counts and reduce the risk of infections; [147] its only curative therapy to date, indicated for patients with a high risk of malignancy, is HSCT [147].

Acquired AA and paroxysmal nocturnal hemoglobinuria (PNH) are diseases belonging to the group of bone marrow failure syndromes and should be considered as two distinct manifestations of a common pathophysiology, but that are interrelated. They frequently affect young patients, although the age distribution for AA shows a bimodal trend with a peak in young adults, while the average age of diagnosis in PNH peaks between 30 and 45 years. Both require long-term care and have high morbidity, despite therapeutic advances [148]. Furthermore, patients with AA and PNH have a risk of developing MDS and AML, with a ten-year incidence of 15-20 % and 2-6 %, respectively [149].

Prospective studies demonstrate that between 69 % and 89 % of AA cases are classified as severe or very severe. These cases are associated with higher morbidity and mortality and a higher incidence of infections compared to non-severe AA. Treatment options include HSCT, immunosuppressive therapy, thrombopoietin receptor agonists, and clinical trials, depending on patient age, clinical performance, and donor availability for HSCT [148]. Although HSCT has high overall survival rates, the availability of compatible donors and patient age are obstacles to its widespread implementation [148]. In patients with moderate to severe anemia, periodic transfusions may be necessary, affecting QoL due to time spent on healthcare and the risk of side effects [150]. Complications related to cytopenias (e.g., symptomatic anemia, febrile neutropenia, sepsis, hemorrhages requiring medical intervention) and secondary to transfusions (e.g., iron overload and iron chelation) are common [148,151,152].

A study conducted with 74 patients diagnosed with AA demonstrated a higher frequency and intensity of psychosocial symptoms in these patients. Symptoms included worries and uncertainties about the future, the need for continuous monitoring, insecurity in planning activities, and fear regarding the worsening of their blood levels. Interestingly, the complications frequently feared and highlighted by attending physicians, such as susceptibility to infections and bleeding tendency, had a small impact on the QoL of patients [153].

In PNH, the lack of complement regulatory proteins (CD55 and CD59) leads to intravascular hemolysis, hemoglobinuria, and an increased frequency of thrombotic complications, which are the main cause of death [154,155]. The greatest risk factor for PNH is immune-mediated AA. Factors such as the size of the PNH clone and the degree of hemolysis may correlate with the intensity of symptoms, which range from asymptomatic to significant impairment of QoL [148,154]. Symptoms related to hemolysis episodes include fatigue, back and abdominal pain, headache, dysphagia, and erectile dysfunction [154,155]. Increased hemolysis can occur during acute events such as infection, surgery, and transfusions [155]. The most common symptom in PNH is fatigue, present in 80.9 % of patients, impacting overall well-being, interfering with daily and work activities, cognitive and psychological functions, and causing demotivation, sadness, frustration, and mental exhaustion [155]. Treatment with complement inhibitors is the choice for patients with high hemolytic activity associated with clinical repercussions, bringing patient survival closer to that of healthy individuals of similar ages [148,155]. However, some patients using C5 complement inhibitors still have residual anemia and fatigue, which limit their ability to perform daily activities (e.g., work, school, and hobbies) [154].

A systematic review found no articles on the role of PC in these diseases. Given the complexity of the symptoms presented by patients with bone marrow failure, the possible referral for HSCT, the recurrent risk of hospitalization, and high morbidity and mortality, we suggest integrating PC similarly to what is recommended for acute leukemias. For patients with PNH, we recommend integration according to the presentation of symptoms, such as more severe life-threatening events or unsatisfactory response to the proposed treatment.

### Recommendations

#### Referral criteria for patients with congenital bone marrow failure, acquired aplastic anemia, and paroxysmal nocturnal hemoglobinuria to palliative care

1. Pediatric patients diagnosed with congenital bone marrow failure: due to high morbidity and mortality and physical, psychological, and social complications, we suggest referral to PC centers preferably from the time of diagnosis, or in the case of failure of the therapeutic strategy, indication for recurrent blood transfusions, or referral for allogeneic HSCT. (Level of recommendation: D)
2. Patients diagnosed during hospital admission with severe or very severe AA: extrapolating the evidence of PC to AML patients, we suggest early integration of PC at diagnosis. (Level of recommendation: D)
3. Patients diagnosed with severe or very severe AA undergoing immunosuppressive therapy and outpatient follow-up, requiring recurrent blood transfusions or regular visits to healthcare services. (Level of evidence D)
4. Patients diagnosed with NPH: unsatisfactory response to disease-modifying treatment and persistence of symptoms despite targeted treatment. (Level of recommendation: D)
5. Patients with recurrent unplanned hospitalizations or due to acute events with physical, psychological, or social impact. (Level of recommendation: D)
6. Patients with a high symptom burden, refractory symptoms, moderate to intense psychological or existential distress, or the presence of any of the criteria described in the general criteria for referring hematological patients to PC. (Level of recommendation: D)

## Congenital anemias and hemorrhagic diseases

Sickle cell disease (SCD) is the most prevalent monogenic disorder in the world [2]. It results from a complex pathophysiological process involving tissue damage mediated by ischemia and hemolysis. Patients experience, in addition to the typical picture of intense acute pain episodes, high morbidity resulting from chronic target organ damage (e.g., retina, lungs, heart, brain, kidneys, and bones) [156]. Pain episodes are the main cause of visits to the pediatric emergency room and hospitalizations [157]. Dactylitis affects children between six months and two years of age, while in older children, crises usually occur in the long bones of the lower limbs, thoracic, and abdominal regions [40,157]. Acute chest syndrome is the second most frequent complication and is the leading cause of death; priapism can lead to sexual impotence; while stroke is a medical emergency with a high risk of mortality and serious sequelae, both physical and cognitive-behavioral [157]. Furthermore, children and adolescents may face insomnia, daytime sleepiness, episodes of anxiety, and depressive states due to social isolation resulting from repeated hospitalizations, in addition to physical impairment and the various stigmas associated with SCD [157,158]. While advances in the diagnosis and early management of SCD complications have increased survival rates, this population now faces a prolonged burden of chronic pain and comorbidities. These factors lead to significant impairment of QoL, characterized by motor limitations and high rates of absenteeism from occupational and educational environments [159]. Clinical complications interfere not only with the patient's life but also with that of their families. Parents or caregivers are subjected to high levels of stress and sometimes manifest mental health problems [157,158].

A study conducted by Toro et al. at a leading Brazilian institution demonstrated that patients with SCD present a high burden of limiting symptoms, including fatigue, depression, hyporexia, and chronic pain [160]. Furthermore, the presence of other comorbidities and social and economic factors, particularly low family income and low education, negatively impacts symptom scores. The prevalence of exposure to interpersonal episodes of violence (physical, emotional, or sexual) is increased and is associated with a higher frequency of chronic pain episodes, regular opioid use, depression, and anxiety [161]. Inadequate analgesic treatment can result in hypersensitivity and amplified responses to subsequent pain episodes, lack of trust in health services, and absenteeism [159]. Recurrent pain episodes and consequent social isolation, associated with cognitive impairment resulting from ischemic stroke events, lead to higher rates of depression and anxiety [162,163]. Sometimes, chronic complications can lead to an unpredictable decline in health conditions, causing patients and, occasionally, family members, to make complex decisions, such as defining end-of-life care [164].

In childhood SCD, some randomized studies, which specifically analyzed pain management, should be considered, since pain relief is understood within the pillars of PC [165,166]. Strategies such as CBT for pain through digital intervention in adolescents aged 12 to 18 years significantly reduced the average intensity of pain and the number of days with pain over six months when compared to the educational control group [143,158,165, 166, 167]. Individualized pain management plans and biofeedback programs can significantly reduce hospital stays and readmission rates, as well as generate substantial savings in hospital costs [168]. Al Zahrani et al. demonstrated that systematic multidisciplinary team assistance for patients with SCD and a history of opioid abuse, by incorporating educational activities, access to PC or pain clinics, rational opioid protocols, and chemical dependency approaches, was able to reduce costs and emergency service visits while significantly decreasing opioid consumption [169]. The combination of psychological treatments can reduce the intensity of chronic pain and disability [170]. A systematic review found that some non-pharmacological interventions for pain management (CBT and biofeedback), both in outpatient and home settings, and during hospital stays (yoga sessions, massage, and virtual reality), significantly reduced the need for analgesics and contributed to more holistic patient care [167].

Individuals with SCD and their families deal with repeated losses throughout their lives, such as physical functionality, the ability to provide for their family, and absenteeism from work/school/family life. The grief of these losses needs recognition and support [25]. Despite the evidence, it is known that patients with SCD have less access to PC [171] and, given the high complexity of the symptoms, most professionals are not accustomed to treating them adequately, resulting in unsatisfactory control of acute and chronic pain, inadequate management of psychosocial and spiritual issues, and a lack of advanced end-of-life care planning [162].

Thalassemias are a heterogeneous group of hereditary diseases that result in distinct phenotypes depending on the partial decrease or absence of synthesis of alpha or beta chains of hemoglobin. Patients may be asymptomatic, with only the thalassemia trait present, or they may present with severe clinical manifestations, including death or severe anemia, requiring regular transfusion regimens and iron overload, with HSCT being the only curative treatment option [40].

Coagulopathies encompass qualitative and quantitative alterations in coagulation factors, potentially leading to mild to severe bleeding disorders depending on the degree of factor deficiency. Bleeding manifestations can be frequent and spontaneous, and, in hemophilia, they occur mainly in the joints, causing deformities and physical sequelae, as well as acute and chronic pain. From birth, there is a high risk of severe bleeding. Currently, prophylaxis and immunotolerance are part of the treatment and monitoring of these patients [172]. Hereditary deficiencies of factors I (fibrinogen), II, V, VII, X, XI, and XIII, as well as combined deficiencies of factors such as V and VIII, are rare, but these disorders can cause potentially fatal bleeding episodes, such as intracranial hemorrhages, especially in the neonatal period [172].

Except for the limited evidence of the applicability of PC in patients with SCD, our systematic review found no articles on PC in other benign hematological diseases, also known as classic hematological diseases. These include a range of different clinical manifestations and the possibility of chronicity, with substantial physical and psychosocial sequelae, such as fatigue, pain secondary to post-thrombotic syndrome, chronic arthropathy, cognitive disorders, and physical injuries from ischemic and/or hemorrhagic strokes, cardiovascular events, organ dysfunction, frequent blood transfusions and intravenous infusions, and occasional hospitalizations, with a consequent impact on the QoL of the patient and their family. PC should, if possible, be integrated into early childhood and continued throughout life, offering support to the patient and their families, assisting them in navigating the complexities of the disease and in making decisions about its treatment, focusing on education about the pathology and its prognostic limitations, genetic counseling, defining a therapeutic plan, controlling physical and psychological symptoms, and assisting in modifying environmental factors that impact treatment (e.g., social, educational, pedagogical, and economic vulnerability factors) [25]. PC can facilitate advanced care planning, assist during hospitalizations, and help align patient expectations with clinical reality. PC also plays a critical role in supporting the complex transition from pediatric to adult-centered care.

### Recommendations

#### Criteria for referring patients with sickle cell disease, thalassemia, congenital anemias, and hemostasis disorders to palliative care

1. Patients with symptoms that are difficult to manage in their different dimensions (physical, social, psychological, and spiritual) and that are refractory to interventions by the referring care team. (Level of recommendation: D)
2. Patients with chronic organ dysfunction or progressive multi-organ impairment; physical and/or cognitive sequelae, progressive loss of functionality, and impairment of QoL. (Level of recommendation: D)
3. Patients with high transfusion needs/chronic transfusions or who require regular visits to health services. (Level of recommendation: D)
4. Patients with recurrent unplanned hospitalizations or hospitalizations due to acute events with physical, psychological, or social impact. (Level of recommendation: D)
5. Patients with a high symptom burden, chronic pain, refractory symptoms, moderate to intense psychological or existential distress, or the presence of any of the criteria described in the general referral criteria of hematological patients to PC. (Level of recommendation: D)

### Final considerations

Although in recent years there has been a growth in publications and studies on PC in hematology, scientific evidence is still scarce. The integration between PC and hematology seeks more effective symptom control, adaptation, and rehabilitation of the patient in the face of functional and social losses; family support in the face of illness and bereavement; alignment of care objectives with the patient's values; and the development of advanced end-of-life care planning [173]. Despite this panorama, the term ‘palliative care’ is frequently misunderstood by healthcare professionals as synonymous with end-of-life care, or as only offered when there are no more disease-modifying treatment options, thereby delaying PC referrals and hindering patients' understanding of the importance and scope of this care. A public opinion survey in the United States showed that most laypeople have never heard the word ‘palliative’ and do not know what it means. On the other hand, as soon as they learn more about PC, they become interested in receiving it for themselves or a loved one [174,175].

Internships in PC during hematology training or continuing education programs are strategies to expand the competencies and skills of hematologists in primary PC, since these are critical components of care for a large proportion of hematological patients. Specialist-level involvement in PC can be restricted to more complex scenarios, being episodic and intermittent, or otherwise continuous, longitudinal, and co-managed [173,174].

Therefore, we believe that the publication of this consensus will allow for the expansion of the discussion on the interface between PC and Hematology, inspiring the integration of these specialties and the search for answers to so many questions and doubts for better care of hematological patients.

## Authors' contributions

All authors contributed to the literature review, development of recommendations, and writing of the text. APSF and PMC created the figures, edited the complete text, and submitted the final version for approval by the other authors.

## Conflicts of interest

The authors declare no conflicts of interest related to this topic.
